# Alpha-smooth muscle actin-positive cancer-associated fibroblasts secreting osteopontin promote growth of luminal breast cancer

**DOI:** 10.1186/s11658-022-00351-7

**Published:** 2022-06-11

**Authors:** Anna Muchlińska, Anna Nagel, Marta Popęda, Jolanta Szade, Magdalena Niemira, Jacek Zieliński, Jarosław Skokowski, Natalia Bednarz-Knoll, Anna J. Żaczek

**Affiliations:** 1 Laboratory of Translational Oncology, Intercollegiate Faculty of Biotechnology, Medical University of Gdansk, 80-211 Gdansk, Poland; 2 Department of Pathomorphology, Medical University of Gdansk, 80-214 Gdansk, Poland; 3 Clinical Research Centre, Medical University of Bialystok, 15-276 Bialystok, Poland; 4 Department of Surgical Oncology, Medical University of Gdansk, 80-214 Gdansk, Poland; 5 Biobanking and Biomolecular Resources Research Infrastructure Poland (BBMRI.PL), 80-211 Gdansk, Poland

**Keywords:** Cancer-associated fibroblast, Luminal breast cancer, Alpha-smooth muscle actin, Osteopontin

## Abstract

**Background:**

Cancer-associated fibroblasts (CAFs) have been shown to support tumor development in a variety of cancers. Different markers were applied to classify CAFs in order to elucidate their impact on tumor progression. However, the exact mechanism by which CAFs enhance cancer development and metastasis is yet unknown.

**Methods:**

Alpha-smooth muscle actin (α-SMA) was examined immunohistochemically in intratumoral CAFs of nonmetastatic breast cancers and correlated with clinicopathological data. Four CAF cell lines were isolated from patients with luminal breast cancer (lumBC) and classified according to the presence of α-SMA protein. Conditioned medium (CM) from CAF cultures was used to assess the influence of CAFs on lumBC cell lines: MCF7 and T47D cells using Matrigel 3D culture assay. To identify potential factors accounting for promotion of tumor growth by α-SMA^high^ CAFs, nCounter PanCancer Immune Profiling Panel (NanoString) was used.

**Results:**

In luminal breast cancer, presence of intratumoral CAFs expressing high level of α-SMA (13% of lumBC group) correlated with poor prognosis (*p* = 0.019). In in vitro conditions, conditioned medium obtained from primary cultures of α-SMA-positive CAFs isolated from luminal tumors was observed to enhance growth of lumBC cell line colonies in 3D Matrigel, in contrast to CM derived from α-SMA-negative CAFs. Multigene expression analysis indicated that osteopontin (OPN) was overexpressed in α-SMA-positive CAFs in both clinical samples and in vitro models. OPN expression was associated with higher percentage of Ki67-positive cells in clinical material (*p* = 0.012), while OPN blocking in α-SMA-positive CAF-derived CM attenuated growth of lumBC cell line colonies in 3D Matrigel.

**Conclusions:**

Our findings demonstrate that α-SMA-positive CAFs might enhance tumor growth via secretion of OPN.

**Supplementary Information:**

The online version contains supplementary material available at 10.1186/s11658-022-00351-7.

## Background

Breast cancer (BC) remains one of the leading causes of cancer-related mortality in women, despite advances in tumor prevention, early detection, and treatment [1, 2]. Tumor microenvironment (TME) plays an important role in cancer initiation and progression [3, 4]. Stroma is composed of vascular endothelial cells, pericytes, mesenchymal stem cells, fibroblasts, and various types of infiltrating immune cells that might interact with each other and tumor cells [4]. In most solid tumors, including breast cancer, cancer-associated fibroblasts (CAFs) are predominant cellular elements of the stroma. CAFs constitute a heterogeneous population. Multiple CAF subtypes with separate molecular profiles and different impact on tumor outgrowth have been identified in various types of cancer [5].

Differential expression of markers such as fibroblast activation protein (FAP), vimentin (VIM), fibroblast-specific protein 1 (FSP1), alpha-smooth muscle actin (α-SMA), and platelet-derived growth factor receptor (PDGFR) is characteristic for CAFs [6–8]. Nevertheless, these markers are far from being comprehensive or exclusively expressed by these subtypes of cells. Therefore, spindle-shape cell morphology is still a commonly used way to identify CAFs within the tumor stroma [9]. CAFs were shown to promote cancer metastasis, as well as affect angiogenesis, immunosuppression, and drug resistance via synthesis and remodeling of the extracellular matrix (ECM) and production of growth factors [9–11]. Thus, CAF-targeting treatment aimed at modifying their number, subtype, or features is a plausible strategy of improving patients’ outcome in the future [12, 13].

Several studies have shown the significance of α-SMA-positive CAFs in development and progression of different solid tumors. It was demonstrated that stromal expression of α-SMA correlate with a high number of lymph node metastases [14, 15] and worse clinical outcome in patients with breast cancer [16]. α-SMA-positive CAFs also enhanced angiogenesis and influenced tumor growth in vivo [17], and correlated with higher frequency of cancer stem cells [18]. Of note, depletion of α-SMA-positive CAFs in pancreatic cancer suppressed immune surveillance by increasing CD4^+^ Foxp3^+^ regulatory T cells (Tregs) in pancreatic cancer [19].

Here, we aimed to better understand the role of α-SMA-positive CAFs in BC, with special interest in their impact on tumor growth and dissemination among molecular subtypes. We studied in detail CAF heterogeneity in luminal breast cancer (lumBC) at both the molecular and functional level. Using primary CAFs derived from tumor tissue of patients with lumBC, we demonstrated how heterogeneous expression of α-SMA in CAFs might determine tumor growth.

## Methods

### Patients

Primary tumors of 108 patients with breast cancer (inclusion criteria: nonlobular histology, stage I–III) treated in the University Clinical Centre in Gdańsk, Poland (2011–2013), were investigated and described previously [20]. The study was approved by the Ethical Committee of the Medical University of Gdansk (NKBBN 94/2017), and informed consent was collected from all participants.

### Immunohistochemistry

Preparation of tissue microarrays (TMA) containing primary breast cancer tissues and staining for ER, PgR, HER2, and Ki67 were performed as previously described [21]. α-SMA (mouse monoclonal antibody, clone 1A4, Dako Agilent, Santa Clara, CA, USA) and EpCAM (mouse monoclonal antibody, clone Ber-EP4, Dako Agilent) were stained and detected with EnVision FLEX Dako Autostainer (Dako Agilent). SNAIL and OPN staining were performed manually using SNAIL mouse monoclonal antibody (clone 2G11, Novus Biologicals, Centennial, CO, USA, dilution 1:100) and OPN rabbit polyclonal antibody (Abcam, Cambridge, UK, dilution 1:1600) for 60 min at room temperature.

For α-SMA, EpCAM, and SNAIL, staining intensity (0, negative; 1, weak; 2, intermediate; 3, strong) and percentage (0–100%) of the stained stromal (α-SMA) or tumor cells (EpCAM, SNAIL) in the total number of stromal or tumor cells, respectively, were determined; index score was calculated as the percentage of positive cells multiplied by intensity, resulting in a score 0–300. For OPN, only the intensity (0, negative; 1, weak; 2, intermediate; 3, strong) of staining in stroma cells was determined. The overall score corresponding to one patient was established. α-SMA high status was conferred to samples with index score higher than the upper quartile (Q3) of the whole group.

### Clinical data analysis

Kaplan–Meier survival curves presenting overall survival (OS) in patients with low versus high α-SMA were compared using log-rank (Mantel–Cox) test. Hazard ratios (HR) with 95% confidence intervals (95% CI) were computed using Cox proportional hazards regression. The chi-squared test or Fisher’s exact test were used to examine the distribution of α-SMA protein status among clinicopathological features (stage, grade, lymph node status). Differences in protein levels between groups were analyzed using Mann–Whitney *U* test. *p*-Values ≤ 0.05 were considered statistically significant. All the analyses were performed using IBM SPSS Statistics version 27 licensed for the University of Gdańsk.

### Isolation of CAFs from breast cancer

CAFs were isolated from tumor samples of four patients with lumBC (CAF2, CAF3, and CAF4 were isolated from patients with luminal B breast cancer, whereas CAF1 from luminal A) obtained from the Department of Surgical Oncology, Medical University of Gdansk (2017–2018) after patients’ written informed consent. Each sample (1–3 cm) was collected by an experienced surgeon in aseptic conditions and transferred into DMEM (HyClone, GE Healthcare, Chicago, IL, USA) supplemented with 10% fetal bovine serum (FBS) (HyClone, GE Healthcare) and antimycotic/antibiotic mix (Sigma Aldrich, Saint Louis, MO, USA). Tissue sections were stored in 2–8 °C and transferred to the Laboratory of Translational Oncology where further processing started no later than 4 h after resection. Tissue sections were washed with 1× PBS, minced, and digested enzymatically in 0.35 mg/ml collagenase (Sigma Aldrich) and 0.35 mg/ml hyaluronidase (Sigma Aldrich) solution in 1× PBS for 1 h with rotation at 37 °C and 5% CO_2_. Disintegrated tissues were centrifuged at 400*g* for 5 min, and the pellet was transferred to a cell culture dish. CAFs were separated using serial trypsinization method. After two to three passages, CAFs were controlled for spindle-shape morphology and presence of CAFs markers VIM and α-SMA, and absence of tumor marker E-cadherin (E-cad).

### Cell culture

Primary CAFs were cultured in DMEM (HyClone, GE Healthcare) supplemented with 10% FBS (HyClone, GE Healthcare) at 37 °C, 5% CO_2_. MCF7 (HTB-22), T47D (CRL-2865), and BJ (CRL-2522) cells were purchased from the American Tissue Culture Collection (ATCC, Manassas, VA, USA). Cells were cultured in DMEM supplemented with 10% FBS and were routinely tested for mycoplasma contamination. For all cell lines, the same batch of FBS was used. Conditioned medium (CM) was obtained from cultures of isolated CAFs and fibroblast cell line, BJ. When cells reached 80% confluency, the medium was changed, and conditioned medium was collected after 72 h.

### CAF characterization

#### Immunofluorescence staining

Cells were seeded on sterilized cover glass and after 24 h were fixed and permeabilized using a methanol–acetone mix for 15 min. For blocking, 5% BSA in PBS was used. Primary antibodies were diluted in Antibody Diluent (Dako Agilent) and incubated with cells for 30 min. Anti-α-SMA (mouse monoclonal, clone 1A4, Dako Agilent, dilution 1:1), anti-vimentin (rabbit polyclonal, Novus Biologicals, dilution 1:1000), and anti-E-cadherin (mouse monoclonal, clone 36, BD, Franklin Lakes, NJ, USA, dilution 1:2000) antibody was used for CAF characterization. As secondary antibodies, anti-rabbit IgG DyLight 594 and anti-mouse IgG DyLight 488 were used (Thermo Fisher Scientific, Waltham, MA, USA; dilution 1:2000). Imaging was performed using an Olympus IX83 fluorescent microscope and CellSens Imaging Software (Olympus Life Science, Waltham, MA, USA).

#### Western blot

Cell lysate was prepared using RIPA buffer (Sigma Aldrich), then protein concentration was measured with a BCA assay kit (Thermo Fisher Scientific). Proteins were separated using 12% polyacrylamide TGX gels (Bio-Rad, Hercules, CA, USA) and transferred onto the PVDF membrane by semi-dry transfer (Bio-Rad). Anti-α-SMA (mouse monoclonal, clone 1A4, Dako Agilent, dilution 1:1), anti-vimentin (rabbit polyclonal, Novus Biologicals, dilution 1:2000), and anti-E-cadherin (mouse monoclonal, clone 36, BD, dilution 1:500) antibodies were used for detection. Appropriate, secondary anti-rabbit and anti-mouse HRP-conjugated antibodies were used (Sigma Aldrich, dilution 1:100,000).

#### ELISA

Osteopontin concentration (ng/ml) in CAFs and BJ-CM was evaluated using human osteopontin (OPN) Quantikine ELISA (R&D Systems, Minneapolis, MN, USA) according to the manufacturer’s protocol. Each sample was assayed in triplicate. Concentration was quantified by measuring the absorption at 450 nm with a microplate reader (Synergy H1, BioTek, USA).

### 3D culture in Matrigel

LumBC cell lines MCF7 and T47D were used to analyze influence of CAFs and BJ-CM on colony growth in 3D culture (Corning Matrigel). A total of 2 × 10^3^ tumor cells resuspended in DMEM were mixed with Matrigel 1:1 and placed into 12-well tissue culture plates. Three-dimensional cultures were then covered with CM from CAFs, BJ, and DMEM as a control, exchanged every third day. For experiments aiming at OPN neutralization, OPN-neutralizing antibody (R&D Systems) diluted 3 μg/ml was used. Tumor-cell colony growth was quantified by measuring the area of at least 40 colonies using Olympus IX83 microscope (10× magnification) and ImageJ software. Data were presented as mean relative to DMEM ± standard deviation (SD) from at least three independent experiments. Comparative data were analyzed with the unpaired Student’s *t*-test using the IBM SPSS statistics software. *p*-Values ≤ 0.05 were considered statistically significant.

### Multigene expression analysis

#### RNA extraction

RNA was isolated from CAFs using RNeasy Mini Kit (Qiagen, Hilden, Germany) according to the manufacturer’s protocol. RNA concentration and purity were determined using NanoDrop 1000 spectrophotometer (Thermo Scientific, Wilmington, DE, USA). RNA extraction from lumBC samples (formalin-fixed paraffin embedded specimens) was performed as described [22].

#### nCounter gene expression assay

The CAFs and clinical samples-derived RNA samples were analyzed in separate batches. RNA extracted from CAFs (300 ng) was subjected to expression profiling with nCounter PanCancer Immune Profiling Panel (NanoString Technologies, Seattle, WA, USA) according to the manufacturer’s procedures for hybridization, detection, and scanning. Analysis of RNA extracted from clinical samples was processed as described [22].

#### NanoString data processing

The CAFs and clinical samples RNA data were processed in separate batches. For each sample, background correction and normalization were performed using nSolver 4.0 software, as previously described [22]. Data were normalized according to the global mean of the counts of positive controls included in the assay and the most stably expressed housekeeping genes, 18 (SD range 2.9–66.9 counts) and 4 (SD range 173.5–228.4 counts) in the CAFs and clinical samples datasets, respectively. Following normalization, low-expression genes (log_2_ mean count in all samples < 4 for CAFs dataset and < 6 for FFPE dataset) were excluded, leaving 320 and 584 transcripts in CAFs and FFPE dataset, respectively. Genes differentiating between α-SMA^high^ and α-SMA^low^ samples were selected on the basis of log_2_ fold change (log2FC) calculated for the median normalized counts of each probe in compared groups. Genes with log2FC > 1 were considered upregulated; genes with log2FC < −1 were considered downregulated in the α-SMA^high^ group.

## Results

### α-SMA-positive CAFs are associated with poor prognosis and more aggressive phenotype of tumor cells in patients with lumBC

**Fig. 1:**
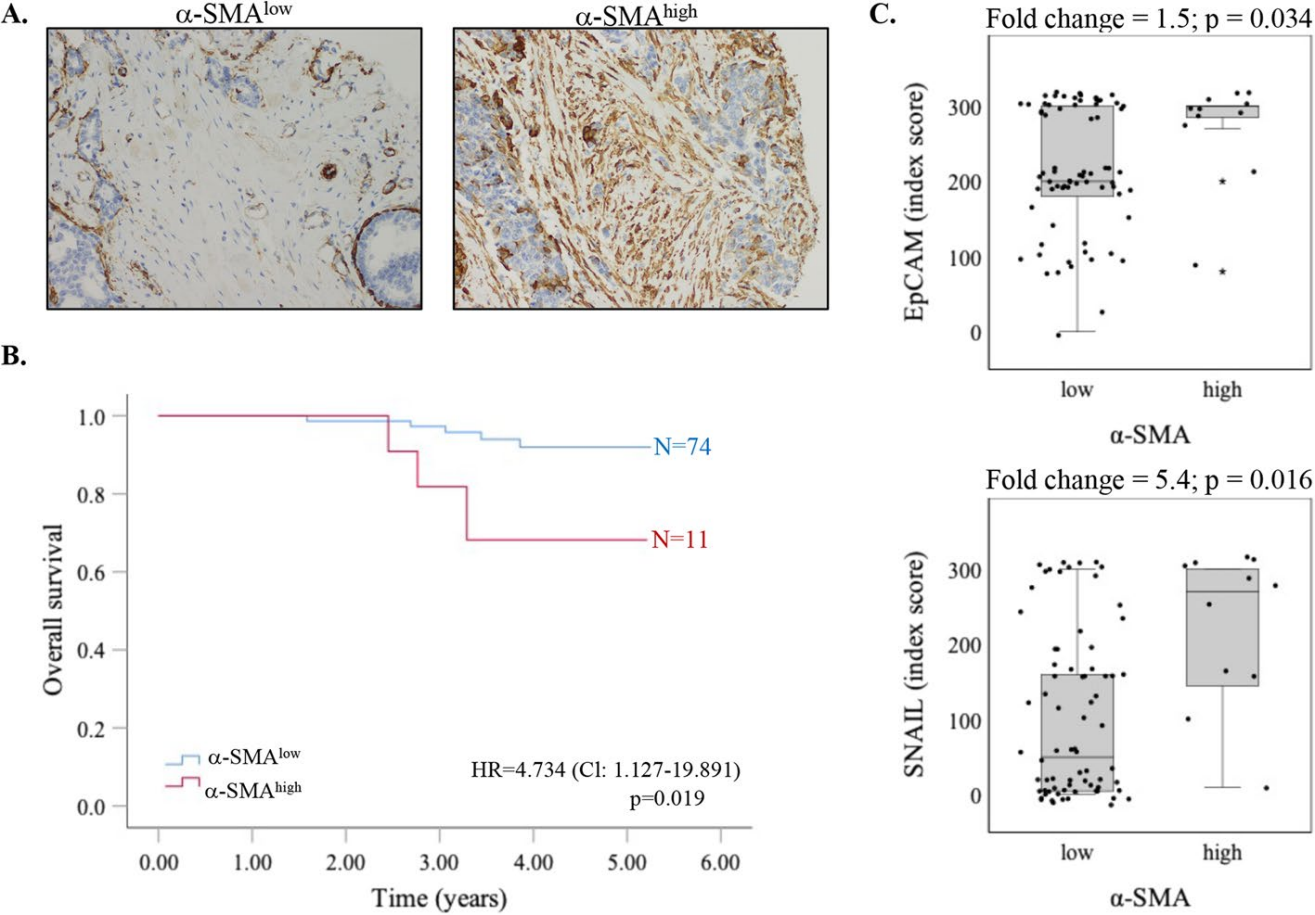
α-SMA-positive CAFs correlate with poor prognosis and more aggressive phenotype of tumor cells in lumBC. **A** Representative staining of α-SMA in TME of breast cancer samples, magnification 20×. **B** Overall survival (OS) in patients with luminal BC according to α-SMA protein level in CAFs. Hazard ratios (HR) with 95% confidence intervals (95% CI) were computed using Cox proportional hazards regression. **C** EpCAM and SNAIL expression in patients with low versus high α-SMA. Mann–Whitney *U* test was applied

α-SMA was evaluated immunohistochemically in fibroblast-like stromal cells of 108 nonmetastatic primary breast tumors (Fig. 1A). Staining was informative for 106 tumors, all of which showed expression of α-SMA in CAFs; importantly, there were considerable differences in the percentage of positive cells (5–90% cells of fibroblast-like morphology) and staining intensity (moderate in 31.7%, high in 68.3% of samples; no weak staining was observed).

For further analysis, patients were divided into two groups, α-SMA^low^ (*n* = 93) and α-SMA^high^ (*n* = 13), according to the upper quartile of the α-SMA staining index (equal 240). The distribution of α-SMA status in CAFs (low versus high) was compared with clinicopathological data, including dissemination status, and molecular data. There was no correlation between α-SMA level and stage, grade, lymph node status, or circulating tumor cell (CTC) presence and phenotype (Additional file 1: Table S1) in the whole analyzed cohort.

Interestingly, only in the lumBC group, patients with α-SMA^high^ CAFs had significantly poorer overall survival rate (*p* = 0.019, log-rank; Fig. 1B). However, no further correlations with clinicopathological data or dissemination status were found in this subgroup (Additional file 1: Table S2). In terms of molecular markers evaluated immunohistochemically, α-SMA^high^ CAFs correlated positively with SNAIL (*p* = 0.034) and EpCAM (*p* = 0.016) protein levels (Fig. 1C), pointing to more aggressive phenotype, while no correlation was observed for other previously characterized proteins, i.e., SLUG, CXCR4, and TWIST [21] (data not shown).

### α-SMA-positive CAFs increase outgrowth of breast cancer cell lines

**Fig. 2:**
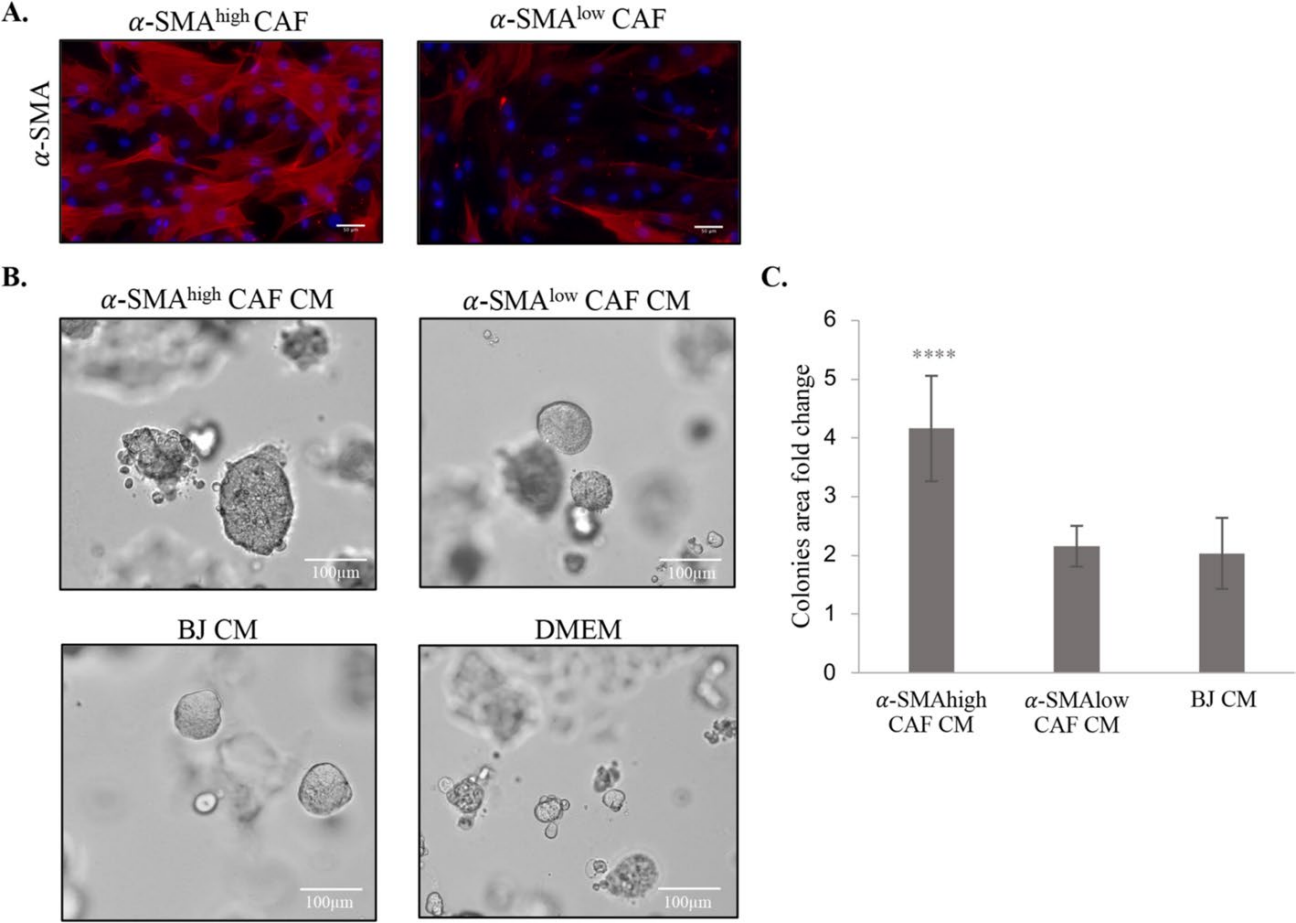
Characterization of CAFs isolated from patients with BC. **A** Representative pictures of immunofluorescence staining for α-SMA (red) in α-SMA^low^ and α-SMA^high^ CAFs; DAPI (blue) was used for nuclei counterstaining. **B** Representative pictures of MCF7 cells growing in 3D-Matrigel cultures, when treated with conditioned medium from α-SMA^low^ or α-SMA^high^ CAFs, and BJ or DMEM as a control. **C** Quantification of colony area fold change. Colony area was determined with ImageJ software. The values presented are mean ± SD from three independent experiments (*n* = 2 technical replicates). Unpaired Student’s *t*-test test was applied, ***p < 0.0001 calculated versus control (DMEM)

To confirm adverse effect of α-SMA CAFs on lumBC, CAFs (*n* = 4) were isolated from primary lumBC tissue specimens and tested on their impact on breast cancer cell lines in vitro. After three passages, isolated CAFs were analyzed for the presence of CAF- and tumor-related markers by immunofluorescent staining and western blot along with BJ normal fibroblasts and MCF7 cell line serving as controls. All obtained CAF cell lines stained positively for VIM and negatively for E-cad and presented spindle-shape morphology typical for fibroblasts (Additional file 2: Fig. S1A, B). On the basis of the α-SMA immunofluorescence staining, CAF cell lines were classified as α-SMA^high^ (CAF3 and CAF4), with the majority of α-SMA-positive cells (i.e., > 90%), or α-SMA^low^ (CAF1 and CAF2), with the majority of α-SMA-negative cells (i.e., > 80%) (Fig. 2A).

CM collected from the established CAFs was applied to lumBC cell lines MCF7 and T47D in 3D Matrigel assay. CM from α-SMA^high^ CAFs significantly increased MCF7 cells colonies growth in Matrigel (colony area fold change compared with control equaled 2.16 for α-SMA^low^ CAFs versus 4.16 for α-SMA^high^ CAFs, *p* = 0.0001) (Fig. 2B, C). Similar observations were obtained for another lumBC cell line, T47D (Additional file 3: Fig. S2).

As these results suggested that α-SMA^high^ CAFs CM promote cancer cell proliferation, we analyzed phosphorylation of two nodal proteins governing pathways activating cell proliferation, AKT and ERK. However, no significant differences were observed in the expression and phosphorylation of those proteins when MCF7 cells were cultured in 2D and treated with CM from α-SMA^high^ or α-SMA^low^ CAFs (data not shown).

Since in clinical material α-SMA^high^ CAFs correlated with higher expression of SNAIL and EpCAM, we performed qPCR expression analysis of these genes in lumBC cell lines treated with CAFs CM. CM from α-SMA^high^ CAFs did not induce expression of SNAIL and EpCAM in lumBC cells (data not shown).

### Osteopontin is expressed and secreted by α-SMA^high^ CAFs

**Fig. 3:**
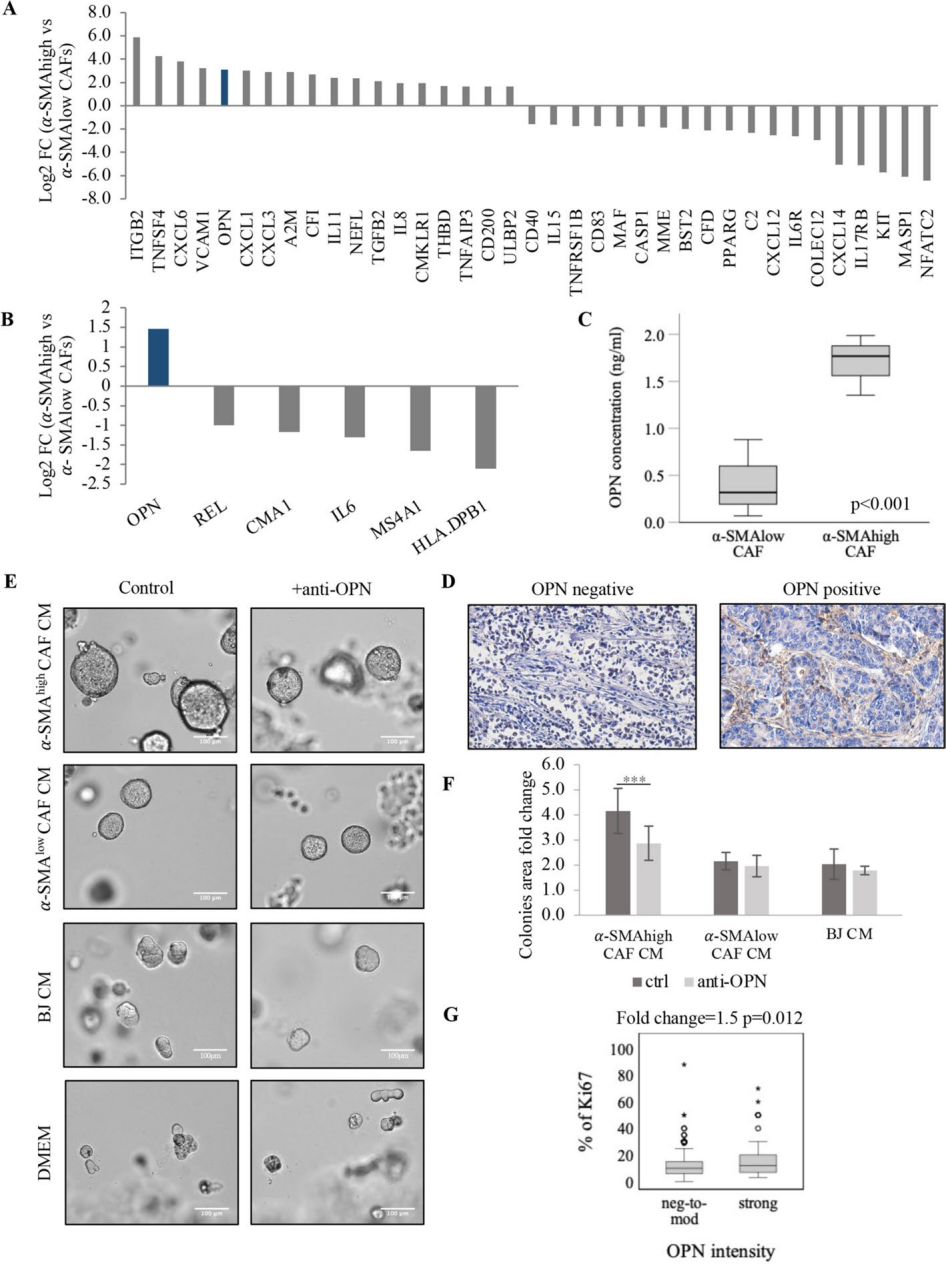
α-SMA^high^ CAF-secreted osteopontin increases tumor growth. **A** nCounter PanCancer Immune Profiling panel analysis of gene expression in patients with α-SMA^high^ versus α-SMA^low^ CAFs (top up- and downregulated genes are presented) and **B** in α-SMA^high^ versus α-SMA^low^ BC; genes with logFC > 1 were considered upregulated, and genes with logFC < −1 were considered downregulated. **C** OPN concentration in α-SMA^high^ versus α-SMA^low^ CAFs measured in CAF medium by ELISA. Graph presents data from three independent experiments (*n* = 2 technical replicates), and error bars show standard deviation. Unpaired Student’s *t*-test test was applied. **D** Representative staining for OPN in CAFs in TME of breast cancer samples, magnification 20×. **E** Representative pictures of MCF7 cells growing in 3D-Matrigel cultures treated with conditioned medium from α-SMA^low^, α-SMA^high^ CAFs, BJ, and DMEM with or without addition of OPN-neutralizing antibodies. **F** Quantification of colony area fold change. Colony area was determined with ImageJ software. The values presented are mean ± SD from three independent experiments (*n* = 2 technical replicates). Unpaired Student’s *t*-test test was applied, ***p < 0.0001 calculated versus control (DMEM). **G** Ki67 percentage in patients with different OPN staining intensity—negative to moderate (neg-to-mod) and strong, evaluated immunohistochemically. Mann–Whitney *U* test was applied

To identify the potential mechanism accounting for promotion of tumor growth by α-SMA^high^ CAFs, multigene expression analysis of four isolated CAF cell lines (two α-SMA^high^ versus two α-SMA^low^) was performed using nCounter PanCancer Immune Profiling Panel (NanoString). Differential gene expression analysis identified 29 genes that were upregulated (logFC > 1, i.e., *ITGB2*, *TNFSF4*, *CXCL6*, *VCAM1*, and *OPN*) in α-SMA^high^ compared with α-SMA^low^ isolated CAFs. They are, e.g., linked with positive regulation of NF-κB transcription factor activity, as revealed by Gene Ontology analysis. In turn, downregulation was noted for 25 genes (logFC < −1, i.e., *NFATC2*, *MASP1*, *KIT*, *IL17RB*, and *CXCL14*) involved in tumor-necrosis-factor-mediated signaling pathway (Fig. 3A, Additional file 4: Table S3).

Then we examined whether the same differences in genes expression were observed in clinical tissue samples from patients with α-SMA^low^ versus α-SMA^high^ lumBC. To highlight the most significant differences between those two groups, scores lower than the lower quartile (Q1) of index score were considered as α-SMA^low^, whereas α-SMA^high^ status was claimed for tissues with scores above the upper quartile (Q3). Only one gene, *OPN*, was significantly upregulated (logFC > 1), and five genes, *HLA-DPB1*, *MS4A1*, *IL6*, *CMA1*, and *REL*, were downregulated (logFC < −1) in patients with α-SMA^high^ (Fig. 3B, Additional file 4: Table S4). Here no interactions were revealed.

Interestingly, *OPN* was the only gene demonstrating upregulation both in α-SMA^high^ CAFs isolated from lumBC and tumor samples of lumBC with α-SMA^high^ CAFs. Consequently, we assessed the secretion of OPN by CAFs in vitro. ELISA assay indeed revealed significantly higher level of OPN protein in the CM from α-SMA^high^ compared with α-SMA^low^ isolated CAFs (FC 4; *p* < 0.001) (Fig. 3C).

In accordance with this observation, when analyzed immunohistochemically in the lumBC tissue material, OPN protein was present in CAF cytoplasm and their surrounding in 51% of samples, mostly scored as weak—1 (41.8%), moderate—2 (38.1%), and strong—3 (20.1%) staining (Fig. 3D). Strong OPN staining was observed in 20% (21/108) of α-SMA^high^ samples when analyzed in individual tumor fragments. OPN expression was also noted in cancer cells, as cytoplasmic or nuclear staining (in 30% and 19% of lumBC samples, respectively). Although in amyotrophic lateral sclerosis OPN-positive fibroblasts were reported to accumulate in perivascular spaces [23], such pattern was not observed in the analyzed breast tumors.

### OPN secreted by α-SMA^high^ CAFs induces lumBC cell colony growth

To further assess the functional significance of CAFs-delivered OPN in lumBC, we performed 3D Matrigel assay. MCF7 and T47D cells were cultured in Matrigel and incubated with different CAF- or BJ-derived CM supplemented or not with OPN-neutralizing antibodies. We observed that addition of OPN-neutralizing antibody decreased colony growth of MCF7 in Matrigel only in cells treated with CM derived from α-SMA^high^ CAFs (colony area fold change compared with control 4.16 for CAFs CM without OPN-neutralizing antibodies versus 2.87 for CM CAFs with OPN-neutralizing antibodies, *p* < 0.001) (Fig. 3E, F). In case of CM from α-SMA^low^ CAFs and BJ cell line, the area of colonies remained unchanged (Fig. 3E, F). Similar results were obtained for another lumBC cell line, T47D (Additional file 3: Fig. S2). In accordance with this observation, OPN strong CAF-associated staining correlated positively with the higher number of Ki67-positive tumor cells assessed immunohistochemically in clinical samples (lumBC, *p* = 0.012, Fig. 3G).

## Discussion

TME and its components such as CAFs play an important role in breast cancer progression [24]. In this study, CAFs were classified according to the presence of α-SMA protein, and their impact on lumBC was investigated in the context of this marker both in clinical samples and in vitro. We showed for the first time that α-SMA^high^ CAFs might stimulate growth of lumBC cancer cells by secreting OPN.

We demonstrated heterogeneous expression of α-SMA in intratumoral CAFs in primary breast cancers. We found that high level of this marker in CAFs was associated with poor prognosis in patients with lumBC. Even though there are studies that link α-SMA^high^ CAFs with worse clinical outcome in breast cancer [16, 25] or specifically in TNBC subtype [26], we report this correlation in lumBC for the first time.

α-SMA^high^ CAFs could potentially influence patients’ outcome by induction or regulation of more aggressive phenotype of cancer cells (characterized, e.g., by increased proliferation, stemness, or epithelial–mesenchymal transition, EMT). In line, CM from α-SMA^high^ CAFs induced the outgrowth of lumBC cell lines in vitro in 3D cultures. However, no induction of phosphorylation of AKT and ERK, proteins involved in cell proliferation, was observed in MCF7 cell line when α-SMA^high^ CAFs CM was used. This could mean that another signaling pathway was involved or α-SMA^high^ CAFs’ positive effect on cancer cell growth occurs only in 3D cultures that more accurately reflect the conditions of tumor growth in vivo. In our cohort of patients with lumBC, significantly higher level of epithelial cell adhesion molecule (EpCAM) and SNAIL were found in α-SMA^high^ tumors. In breast cancer, EpCAM is associated with an unfavorable prognosis in the luminal and basal-like molecular subtypes [27]. There are limited studies correlating CAFs with EpCAM, yet Eberlein et al. demonstrated that tumor cells (non-small-cell lung cancer) with high expression of EpCAM activate CAF-like phenotype in normal fibroblasts through avb6/TGFβ signaling [28]. Thus, potential interaction between EpCAM-positive breast cancer cells and α-SMA^high^ CAFs should be investigated in the future. In case of SNAIL, this transcriptional factor is essential for EMT and induces tumor cell invasion. It has been reported that SNAIL level is upregulated in tumor cells by CAFs in breast cancer [29]. Interestingly, EpCAM could also contribute to the TGF‐β1-induced EMT in lumBC cell line, MCF-7 [30]. Thus, in our study, elevated EpCAM and SNAIL in tumor cells of patients with α-SMA^high^ CAFs may possibly induce EMT, resulting in increased motility of those cells and giving rationale for worse clinical outcome of those patients. Nevertheless, we did not confirm induction of EMT by α-SMA^high^ CAFs in vitro (data not shown). CM from α-SMA^high^ CAFs did not induce expression of SNAIL and EpCAM in lumBC cells. Such results may be due to performing experiments in 2D culture or EMT induction by those CAFs not being dependent on secretome but rather dependent on direct contact of interacting cells.

To dissect putative factors accounting for α-SMA^high^ CAF-mediated induction of lumBC cell outgrowth and worse prognosis, we analyzed their transcriptome both in clinical samples and in vitro. Gene expression revealed that genes upregulated in α-SMA^high^ CAFs (i.e., *IL6*, *IL1B*, *ITGB2*, *ICAM1*) were linked with positive regulation of NF-κB transcription factor activity, which was previously described by our team as EMT-promoting mechanism in breast cancers [22]. In turn, genes downregulated in α-SMA^high^ isolated CAFs (e.g., *CD40*, *TNFRSF14*, *TNFRSF1B*, *PSMB9*) were implicated in tumor-necrosis-factor-mediated signaling pathway. Interestingly, only *OPN* was upregulated both in α-SMA^high^ CAFs isolated from lumBC and tumor tissue samples from lumBC with α-SMA^high^ CAFs.

OPN is a secreted, integrin-binding phosphoprotein involved in carcinogenesis [31–35]. Of note, most studies report on tumor-derived OPN, and little is known about OPN secreted by CAFs. OPN secreted by tumor cells induced expression of CAF-associated markers (α-SMA and VIM) in mesenchymal stromal cells through upregulation of TGF-β1 [36]. It also plays a key role in reprogramming normal mammary fibroblasts to proinflammatory, tumor-promoting CAFs [37]. In breast cancer, elevated expression of OPN was firstly identified in stroma from patients with poor outcome and in CAFs from MMTV-PyMT breast cancer mouse model [38, 39]. We observed positive OPN staining in cancer cell cytoplasm and nucleus, but mostly OPN was present in CAF cytoplasm and surroundings. Here we showed that α-SMA^high^ CAF-derived OPN enhances tumor growth, and this process may be inhibited by OPN-neutralizing antibodies. In our clinical samples, CAF-associated OPN (i.e., OPN found in cytoplasm and/or in surrounding of CAFs) correlated also with the higher percentage of Ki67-positive tumor cells, which would stay in accordance with lumBC cell outgrowth depending on α-SMA^high^ CAFs-derived OPN-induced tumor outgrowth. One of the previously proposed mechanisms of how stroma-derived OPN impacts cell proliferation and survival was involvement of CD44 and activation of MAPK cascade [40]. Whether the regulation of OPN in α-SMA^high^ CAFs is through one of the known pathways has yet to be determined.

## Conclusions

We showed that α-SMA^high^ CAFs correlate with worse prognosis in lumBC and might be associated with more aggressive phenotype of breast cancer cells (e.g., EMT-related phenotype and/or increased proliferation). OPN secretion might be one of the mechanisms accounting for this phenomenon. However, the other mechanisms accounting for adverse impact of α-SMA^high^ CAFs on lumBC merit further investigation.

## Supplementary Information

**Additional file 1: Table S1.** Distribution of α-SMA protein levels in intratumor CAFs among clinico-pathological features of patients with breast cancer. Chi squared test or Fisher’s exact test (F) was used to analyze correlations. Due to missing data not all numbers sum up to 106. **Table S2.** Distribution of α-SMA protein levels in intratumor CAFs among clinico-pathological features of patients with luminal BC. Chi squared test or Fisher’s exact test (F) was used to analyze correlations. Due to missing data not all numbers sum up to 85.

**Additional file 2: Figure S1.** Characterization of CAFs isolated from BC patients. A. Immunofluorescence staining for E-cadherin (E-cad, red), vimentin (VIM, green), α-SMA (red) and DAPI as a nuclear staining (blue) for the established CAFs cultures and BJ or MCF7 cell lines as a control. B. Western blot analysis of E-cad, VIM, and C. α-SMA protein level in CAFs cultures and BJ or MCF7 cell lines as a control.

**Additional file 3: Figure S2.** 3D Matrigel cultures outgrowth of T47D cells. A. Representative pictures of T47D cells growing in 3D-Matrigel cultures treated with conditioned media from α-SMA^low^, α-SMA^high^ CAFs, BJ and DMEM with or without addition of OPN-neutralizing antibodies. B. Quantification of colonies area fold change. Colonies area was determined with ImageJ software. The values presented are means ± SD from 3 independent experiments (*n* = 2 technical replicates). Unpaired Student’s *t*-test test was applied, **p < 0.001 ***p < 0.0001 calculated vs. control (DMEM).

**Additional file 4: Table S3**. nCounter PanCancer Immune Profiling panel analysis of gene expression in α-SMA^high^ vs. α-SMA^low^ isolated CAFs, genes with logFC > 1 were considered upregulated, genes with logFC < -1 were considered downregulated. **Table S4.** nCounter PanCancer Immune Profiling panel analysis of gene expression in α-SMA^high^ vs. α-SMA^low^ lumBC patients. Genes with logFC > 1 were considered upregulated. genes with logFC < -1 were considered downregulated.

## Data Availability

The datasets generated and/or analyzed during the current study are available in the GEO repository, https://www.ncbi.nlm.nih.gov/geo/query/acc.cgi?acc=GSE180186. The datasets used and/or analyzed during the current study are available from the corresponding author on reasonable request.

## Abbreviations

CAFs: Cancer-associated fibroblasts
α-SMA: Alpha-smooth muscle actin
lumBC: Luminal breast cancer
CM: Conditioned medium
OPN: Osteopontin
BC: Breast cancer
TME: Tumor microenvironment
FAP: Fibroblast activation protein
VIM: Vimentin
FSP1: Fibroblast-specific protein 1
PDGFR: Platelet-derived growth factor receptor
ECM: Extracellular matrix
EpCAM: Epithelial cell adhesion molecule

## References

[1] Siegel RL, Miller KD, Fuchs HE, Jemal A (2021). Cancer statistics, 2021. CA Cancer J Clin.

[2] Sung H, Ferlay J, Siegel RL, Laversanne M, Soerjomataram I, Jemal A (2021). Global cancer statistics 2020: GLOBOCAN estimates of incidence and mortality worldwide for 36 cancers in 185 countries. CA Cancer J Clin.

[3] Li H, Fan X, Houghton J (2007). Tumor microenvironment: the role of the tumor stroma in cancer. J Cell Biochem.

[4] Quail DF, Joyce JA (2013). Microenvironmental regulation of tumor progression and metastasis. Nat Med.

[5] Costa A, Kieffer Y, Scholer-Dahirel A, Pelon F, Bourachot B, Cardon M (2018). Fibroblast heterogeneity and immunosuppressive environment in human breast cancer. Cancer Cell.

[6] Bartoschek M, Oskolkov N, Bocci M, Lövrot J, Larsson C, Sommarin M (2018). Spatially and functionally distinct subclasses of breast cancer-associated fibroblasts revealed by single cell RNA sequencing. Nat Commun.

[7] Lee YT, Tan YJ, Falasca M, Oon CE (2020). Cancer-associated fibroblasts: epigenetic regulation and therapeutic intervention in breast cancer. Cancers.

[8] Sahai E, Astsaturov I, Cukierman E, DeNardo DG, Egeblad M, Evans RM (2020). A framework for advancing our understanding of cancer-associated fibroblasts. Nat Rev Cancer.

[9] Kalluri R (2016). The biology and function of fibroblasts in cancer. Nat Rev Cancer.

[10] Tripathi M, Billet S, Bhowmick NA (2012). Understanding the role of stromal fibroblasts in cancer progression. Cell Adhes Migr.

[11] LeBleu VS, Kalluri R (2018). A peek into cancer-associated fibroblasts: origins, functions and translational impact. Dis Model Mech.

[12] Yoshida GJ, Azuma A, Miura Y, Orimo A (2019). Activated fibroblast program orchestrates tumor initiation and progression; molecular mechanisms and the associated therapeutic strategies. Int J Mol Sci.

[13] Wu F, Yang J, Liu J, Wang Y, Mu J, Zeng Q (2021). Signaling pathways in cancer-associated fibroblasts and targeted therapy for cancer. Signal Transduct Target Ther.

[14] Yazhou C, Wenlv S, Weidong Z, Licun W (2004). Clinicopathological significance of stromal myofibroblasts in invasive ductal carcinoma of the breast. Tumor Biol.

[15] Toullec A, Gerald D, Despouy G, Bourachot B, Cardon M, Lefort S (2010). Oxidative stress promotes myofibroblast differentiation and tumour spreading. EMBO Mol Med.

[16] Yamashita M, Ogawa T, Zhang X, Hanamura N, Kashikura Y, Takamura M (2012). Role of stromal myofibroblasts in invasive breast cancer: stromal expression of alpha-smooth muscle actin correlates with worse clinical outcome. Breast Cancer.

[17] Benyahia Z, Dussault N, Cayol M, Sigaud R, Berenguer-Daizé C, Delfino C (2017). Stromal fibroblasts present in breast carcinomas promote tumor growth and angiogenesis through adrenomedullin secretion. Oncotarget.

[18] Patel AK, Vipparthi K, Thatikonda V, Arun I, Bhattacharjee S, Sharan R (2018). A subtype of cancer-associated fibroblasts with lower expression of alpha-smooth muscle actin suppresses stemness through BMP4 in oral carcinoma. Oncogenesis.

[19] Özdemir BC, Pentcheva-Hoang T, Carstens JL, Zheng X, Wu CC, Simpson TR (2014). Depletion of carcinoma-associated fibroblasts and fibrosis induces immunosuppression and accelerates pancreas cancer with reduced survival. Cancer Cell.

[20] Markiewicz A, Książkiewicz M, Wełnicka-Jaśkiewicz M, Seroczyńska B, Skokowski J, Szade J (2014). Mesenchymal phenotype of CTC-enriched blood fraction and lymph node metastasis formation potential. PLoS ONE.

[21] Markiewicz A, Wełnicka-Jaśkiewicz M, Seroczyńska B, Skokowski J, Majewska H, Szade J (2014). Epithelial-mesenchymal transition markers in lymph node metastases and primary breast tumors—relation to dissemination and proliferation. Am J Transl Res.

[22] Popeda M, Stokowy T, Bednarz-Knoll N, Jurek A, Niemira M, Bielska A (2019). NF-kappa B signaling-related signatures are connected with the mesenchymal phenotype of circulating tumor cells in non-metastatic breast cancer. Cancers.

[23] Månberg A, Skene N, Sanders F, Trusohamn M, Remnestål J, Szczepińska A (2021). Altered perivascular fibroblast activity precedes ALS disease onset. Nat Med.

[24] Lu L, Huang J, Mo J, Da X, Li Q, Fan M (2022). Exosomal lncRNA TUG1 from cancer-associated fibroblasts promotes liver cancer cell migration, invasion, and glycolysis by regulating the miR-524-5p/SIX1 axis. Cell Mol Biol Lett.

[25] Hu G, Xu F, Zhong K, Wang S, Huang L, Chen W (2018). Activated tumor-infiltrating fibroblasts predict worse prognosis in breast cancer patients. J Cancer.

[26] Zhou J, Wang XH, Zhao YX, Chen C, Xu XY, Sun Q (2018). Cancer-associated fibroblasts correlate with tumor-associated macrophages infiltration and lymphatic metastasis in triple negative breast cancer patients. J Cancer.

[27] Soysal SD, Muenst S, Barbie T, Fleming T, Gao F, Spizzo G (2013). EpCAM expression varies significantly and is differentially associated with prognosis in the luminal B HER2+, basal-like, and HER2 intrinsic subtypes of breast cancer. Br J Cancer.

[28] Eberlein C, Rooney C, Ross SJ, Farren M, Weir HM, Barry ST (2015). E-Cadherin and EpCAM expression by NSCLC tumour cells associate with normal fibroblast activation through a pathway initiated by integrin αvβ6 and maintained through TGFβ signalling. Oncogene.

[29] Yu Y, Xiao CH, Tan LD, Wang QS, Li XQ, Feng YM (2014). Cancer-associated fibroblasts induce epithelial–mesenchymal transition of breast cancer cells through paracrine TGF-β signalling. Br J Cancer.

[30] Gao J, Yan Q, Wang J, Liu S, Yang X (2015). Epithelial-to-mesenchymal transition induced by TGF-β1 is mediated by AP1-dependent EpCAM expression in MCF-7 cells: EpCAM involved in EMT. J Cell Physiol.

[31] Coppola D, Szabo M, Boulware D, Muraca P, Alsarraj M, Chambers AF (2004). Correlation of osteopontin protein expression and pathological stage across a wide variety of tumor histologies. Clin Cancer Res.

[32] Rittling SR, Chambers AF (2004). Role of osteopontin in tumour progression. Br J Cancer.

[33] Hao C, Cui Y, Owen S, Li W, Cheng S, Jiang WG (2017). Human osteopontin: potential clinical applications in cancer (Review). Int J Mol Med.

[34] Zhao H, Chen Q, Alam A, Cui J, Suen KC, Soo AP (2018). The role of osteopontin in the progression of solid organ tumour. Cell Death Dis.

[35] Zhang X, Zhang L, Tan X, Lin Y, Han X, Wang H (2018). Systematic analysis of genes involved in oral cancer metastasis to lymph nodes. Cell Mol Biol Lett.

[36] Weber CE, Kothari AN, Wai PY, Li NY, Driver J, Zapf MAC (2015). Osteopontin mediates an MZF1–TGF-β1-dependent transformation of mesenchymal stem cells into cancer-associated fibroblasts in breast cancer. Oncogene.

[37] Sharon Y, Raz Y, Cohen N, Ben-Shmuel A, Schwartz H, Geiger T (2015). Tumor-derived osteopontin reprograms normal mammary fibroblasts to promote inflammation and tumor growth in breast cancer. Cancer Res.

[38] Erez N, Truitt M, Olson P, Hanahan D (2010). Cancer-associated fibroblasts are activated in incipient neoplasia to orchestrate tumor-promoting inflammation in an NF-κB-dependent manner. Cancer Cell.

[39] Xu BJ, Yan W, Jovanovic B, Shaw AK, An QA, Eng J (2011). Microdialysis combined with proteomics for protein identification in breast tumor microenvironment in vivo. Cancer Microenviron.

[40] Luo X, Ruhland MK, Pazolli E, Lind AC, Stewart SA (2011). Osteopontin stimulates preneoplastic cellular proliferation through activation of the MAPK pathway. Mol Cancer Res.

