# Supplementary material for: Alpha-smooth muscle actin-positive cancer-associated fibroblasts secreting osteopontin promote growth of luminal breast cancer
Source: Cell Mol Biol Lett. 2022 Jun 11;27:45. doi: 10.1186/s11658-022-00351-7 (PMC9188043; doi:10.1186/s11658-022-00351-7)
Supplement: Supplementary file 1 — Additional file 1: Table S1. Distribution of α-SMA protein levels in intratumor CAFs among clinico-pathological features of patients with breast cancer. Chi squared test or Fisher’s exact test (F) was used to analyze correlations. Due to missing data not all numbers sum up to 106. Table S2. Distribution of α-SMA protein levels in intratumor CAFs among clinico-pathological features of patients with luminal BC. Chi squared test or Fisher’s exact test (F) was used to analyze correlations. Due to missing data not all numbers sum up to 85. [file 11658_2022_351_MOESM1_ESM.docx]

**Table S1**

Distribution of α-SMA protein levels in intratumor CAFs among clinico-pathological features of patients with breast cancer. Chi squared test or Fisher’s exact test (F) was used to analyze correlations. Due to missing data not all numbers sum up to 106.

| Variable | N | α-SMA^low^ | α-SMA^high^ | p |
| --- | --- | --- | --- | --- |
| **T stage** |  |  |  | 1.00 (F) |
| T1-2 | 100 | 87 (83.7%) | 13 (12.5%) |  |
| T3-4 | 4 | 4 (3.8%) | 0 (0%) |  |
| **Grading** |  |  |  | 0.96 |
| 1 | 14 | 12 (11.4%) | 2 (1.9%) |  |
| 2 | 52 | 46 (43.8%) | 6 (5.7%) |  |
| 3 | 39 | 34 (32.4%) | 5 (4.8%) |  |
| **N stage** |  |  |  | 0.77 (F) |
| 0 | 49 | 42 (40%) | 7 (6.7%) |  |
| 1 | 56 | 50 (54.3%) | 6 (5.7%) |  |
| **CTC presence** |  |  |  | 0.39 (F) |
| 0 | 61 | 57 (67.9%) | 4 (4.8%) |  |
| 1 | 23 | 20 (23.8%) | 3 (3.6%) |  |
| **Molecular type** |  |  |  | 0.07 |
| Luminal A | 35 | 33 (31.1%) | 2 (1.9%) |  |
| Luminal B HER2- | 26 | 23 (21.7%) | 3 (2.8%) |  |
| Luminal B HER2+ | 24 | 18 (17%) | 6 (5.7%) |  |
| Non luminal HER2+ | 5 | 5 (4.7%) | 0 (0%) |  |
| Triple Negative | 16 | 14 (13.2%) | 2 (1.9%) |  |

**Table S2**

Distribution of α-SMA protein levels in intratumor CAFs among clinico-pathological features of patients with luminal BC. Chi squared test or Fisher’s exact test (F) was used to analyze correlations. Due to missing data not all numbers sum up to 85.

| Variable | N | α-SMA^low^ | α-SMA^high^ | p |
| --- | --- | --- | --- | --- |
| Age |  |  |  | 0.69 (F) |
| < 50 years | 17 (20%) | 14 (16.5%) | 3 (3.5%) |  |
| ≥ 50 years | 68 (80%) | 60 (70.6%) | 8 (11.8%) |  |
| T stage |  |  |  | 1.00 (F) |
| T1-2 | 81 (95.3%) | 70 (82.4%) | 11 (12.9%) |  |
| T3-4 | 4 (4.7%) | 4 (4.7%) | 0 (0%) |  |
| Grading |  |  |  | 0.92 |
| 1 | 14 (16.5) | 12 (14.1%) | 2 (2.4%) |  |
| 2 | 51 (60%) | 45 (52.9%) | 6 (7.1%) |  |
| 3 | 20 (23.5%) | 17 (20%) | 3 (3.5%) |  |
| N stage |  |  |  | 1.00 (F) |
| 0 | 41 (48.2%) | 36 (42.4%) | 5 (5.9%) |  |
| 1 | 44 (51.8%) | 38 (44.7%) | 6 (7.1%) |  |
| CTC presence |  |  |  | 1.00 (F) |
| 0 | 48 (69.6%) | 44 (63.8%) | 4 (5.8%) |  |
| 1 | 21 (30.4%) | 19 (27.5%) | 2 (2.8%) |  |
