# Supplementary figures and images for: Alpha-smooth muscle actin-positive cancer-associated fibroblasts secreting osteopontin promote growth of luminal breast cancer
Source: Cell Mol Biol Lett. 2022 Jun 11;27:45. doi: 10.1186/s11658-022-00351-7 (PMC9188043; doi:10.1186/s11658-022-00351-7)

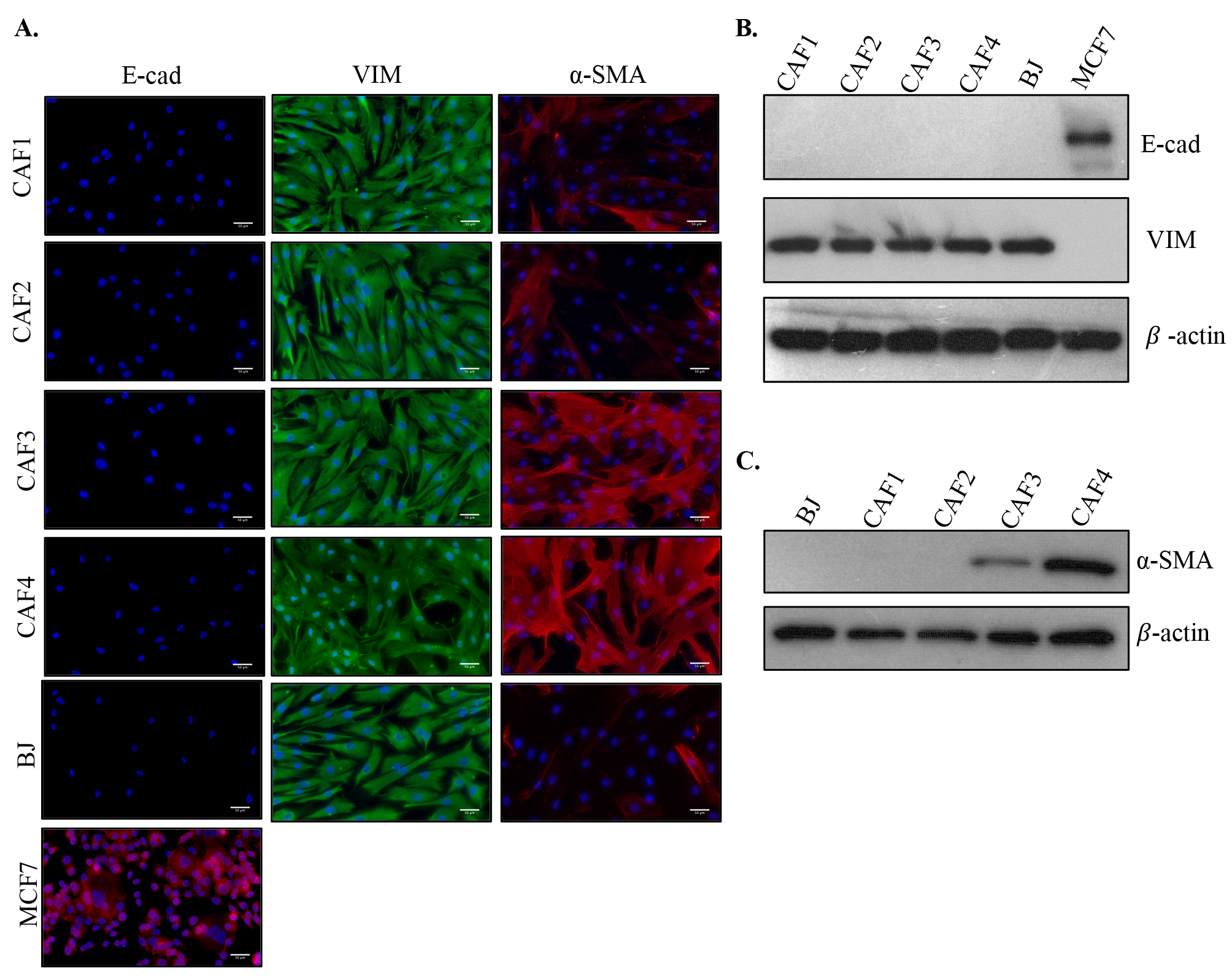

Supplement: Supplementary file 2 — Additional file 2: Figure S1. Characterization of CAFs isolated from BC patients. A. Immunofluorescence staining for E-cadherin (E-cad, red), vimentin (VIM, green), α-SMA (red) and DAPI as a nuclear staining (blue) for the established CAFs cultures and BJ or MCF7 cell lines as a control. B. Western blot analysis of E-cad, VIM, and C. α-SMA protein level in CAFs cultures and BJ or MCF7 cell lines as a control. [file 11658_2022_351_MOESM2_ESM.tif]

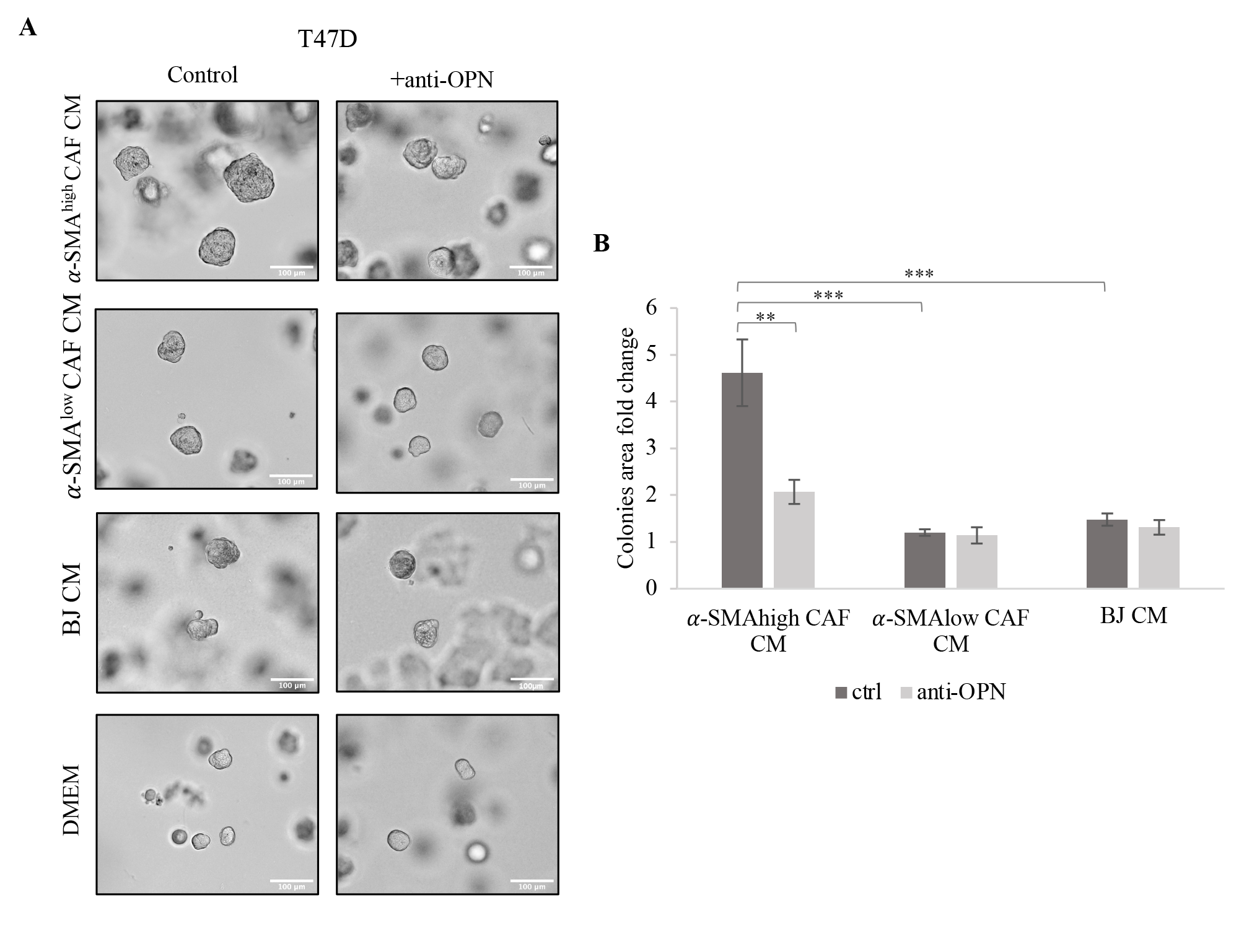

Supplement: Supplementary file 3 — Additional file 3: Figure S2. 3D Matrigel cultures outgrowth of T47D cells. A. Representative pictures of T47D cells growing in 3D-Matrigel cultures treated with conditioned media from α-SMAlow, α-SMAhigh CAFs, BJ and DMEM with or without addition of OPN-neutralizing antibodies. B. Quantification of colonies area fold change. Colonies area was determined with ImageJ software. The values presented are means ± SD from 3 independent experiments (n = 2 technical replicates). Unpaired Student’s t-test test was applied, **p < 0.001 ***p < 0.0001 calculated vs. control (DMEM). [file 11658_2022_351_MOESM3_ESM.tif]
