# Supplementary material for: Alpha-smooth muscle actin-positive cancer-associated fibroblasts secreting osteopontin promote growth of luminal breast cancer
Source: Cell Mol Biol Lett. 2022 Jun 11;27:45. doi: 10.1186/s11658-022-00351-7 (PMC9188043; doi:10.1186/s11658-022-00351-7)
Supplement: Supplementary file 4 — Additional file 4: Table S3. nCounter PanCancer Immune Profiling panel analysis of gene expression in α-SMAhigh vs. α-SMAlow isolated CAFs, genes with logFC > 1 were considered upregulated, genes with logFC < -1 were considered downregulated. Table S4. nCounter PanCancer Immune Profiling panel analysis of gene expression in α-SMAhigh vs. α-SMAlow lumBC patients. Genes with logFC > 1 were considered upregulated. genes with logFC < -1 were considered downregulated. [file 11658_2022_351_MOESM4_ESM.docx]

**Table S3**

nCounter PanCancer Immune Profiling panel analysis of gene expression in α-SMA^high^ vs α-SMA^low^ isolated CAFs, genes with logFC > 1 were considered upregulated, genes with logFC < -1 were considered downregulated.

| **Gene** | **median α-SMA^low^** | **median α-SMA^high^** | **log2FC** |
| --- | --- | --- | --- |
| **UP** | | | |
| ITGB2 | 0.00 | 57.95 | 5.88 |
| TNFSF4 | 32.56 | 648.16 | 4.27 |
| CXCL6 | 66.82 | 941.76 | 3.80 |
| VCAM1 | 5.69 | 60.81 | 3.21 |
| OPN | 6.16 | 60.26 | 3.10 |
| CXCL1 | 145.16 | 1184.91 | 3.02 |
| CXCL3 | 9.00 | 73.88 | 2.90 |
| A2M | 23.04 | 178.17 | 2.90 |
| CFI | 7.11 | 50.86 | 2.68 |
| IL11 | 9.95 | 57.16 | 2.41 |
| NEFL | 9.00 | 49.44 | 2.33 |
| TGFB2 | 44.05 | 192.04 | 2.10 |
| IL8 | 88.68 | 343.43 | 1.94 |
| CMKLR1 | 6.83 | 28.87 | 1.93 |
| THBD | 48.67 | 157.98 | 1.68 |
| TNFAIP3 | 35.04 | 113.59 | 1.67 |
| CD200 | 35.06 | 112.25 | 1.65 |
| ULBP2 | 28.88 | 91.63 | 1.63 |
| IL13RA2 | 191.09 | 523.14 | 1.45 |
| EGR2 | 11.37 | 32.67 | 1.44 |
| IFIT2 | 28.45 | 78.91 | 1.44 |
| IL1A | 27.48 | 74.46 | 1.41 |
| CD9 | 270.62 | 708.64 | 1.39 |
| IL32 | 98.70 | 253.98 | 1.35 |
| IL6 | 237.55 | 576.21 | 1.27 |
| IL1B | 28.43 | 67.48 | 1.22 |
| CXCL2 | 28.99 | 68.06 | 1.20 |
| LRRN3 | 17.53 | 38.85 | 1.10 |
| ICAM1 | 72.84 | 155.49 | 1.08 |
| **DOWN** | | | |
| OAS3 | 24.23 | 11.63 | -1.00 |
| IL7R | 475.92 | 236.42 | -1.01 |
| IL1R1 | 956.96 | 465.83 | -1.04 |
| PSMB9 | 274.79 | 125.82 | -1.12 |
| TNFRSF14 | 132.43 | 59.90 | -1.13 |
| DPP4 | 1332.82 | 530.04 | -1.33 |
| CD40 | 102.95 | 33.45 | -1.59 |
| IL15 | 96.66 | 30.45 | -1.63 |
| TNFRSF1B | 32.49 | 8.95 | -1.75 |
| CD83 | 27.92 | 7.54 | -1.76 |
| MAF | 58.29 | 16.37 | -1.77 |
| CASP1 | 129.89 | 37.13 | -1.78 |
| MME | 639.54 | 174.71 | -1.87 |
| BST2 | 36.78 | 8.48 | -1.99 |
| CFD | 157.55 | 35.48 | -2.12 |
| PPARG | 28.96 | 5.82 | -2.14 |
| C2 | 64.63 | 12.22 | -2.31 |
| CXCL12 | 7014.29 | 1212.54 | -2.53 |
| IL6R | 51.98 | 7.56 | -2.63 |
| COLEC12 | 526.04 | 66.60 | -2.96 |
| CXCL14 | 32.58 | 0.00 | -5.07 |
| IL17RB | 33.55 | 0.00 | -5.11 |
| KIT | 52.02 | 0.00 | -5.73 |
| MASP1 | 67.02 | 0.00 | -6.09 |
| NFATC2 | 84.60 | 0.00 | -6.42 |

**Table S4**

nCounter PanCancer Immune Profiling panel analysis of gene expression in α-SMA^high^ vs α-SMA^low^ lumBC patients. genes with logFC > 1 were considered upregulated. genes with logFC < -1 were considered downregulated.

| **Gene** | **median α-SMA^low^** | **median α-SMA^high^** | **p-value** | **log2FC** |
| --- | --- | --- | --- | --- |
| **UP** | | | | |
| OPN | 2812 | 7731 | 0.03 | 1.46 |
| **DOWN** | | | | |
| REL | 941 | 471 | 0.01 | -1.00 |
| CMA1 | 198 | 88 | 0.03 | -1.17 |
| IL6 | 115 | 47 | 0.03 | -1.30 |
| MS4A1 | 440 | 141 | 0.05 | -1.65 |
| HLA.DPB1 | 27001 | 6274 | 0.01 | -2.11 |
